# PCDH7 interacts with GluN1 and regulates dendritic spine morphology and synaptic function

**DOI:** 10.1038/s41598-020-67831-8

**Published:** 2020-07-02

**Authors:** Yuanyuan Wang, Meghan Kerrisk Campbell, Irene Tom, Oded Foreman, Jesse E. Hanson, Morgan Sheng

**Affiliations:** 1 Department of Neuroscience, Genentech Inc., South San Francisco, CA 94080 USA; 2 Department of OMNI-Biomarker, Genentech Inc., South San Francisco, CA 94080 USA; 3 Department of Pathology, Genentech Inc., South San Francisco, CA 94080 USA; 4 Present Address: Alkahest Inc., San Carlos, CA 94070 USA; 5 Present Address: Stanley Center for Psychiatric Research, Broad Institute of MIT and Harvard, Cambridge, MA 02142 USA

**Keywords:** Membrane proteins, Cadherins, Cellular neuroscience, Spine structure, Synaptic transmission

## Abstract

The N-terminal domain (NTD) of the GluN1 subunit (GluN1-NTD) is important for NMDA receptor structure and function, but the interacting proteins of the GluN1-NTD are not well understood. Starting with an unbiased screen of ~ 1,500 transmembrane proteins using the purified GluN1-NTD protein as a bait, we identify Protocadherin 7 (PCDH7) as a potential interacting protein. PCDH7 is highly expressed in the brain and has been linked to CNS disorders, including epilepsy. Using primary neurons and brain slice cultures, we find that overexpression and knockdown of PCDH7 induce opposing morphological changes of dendritic structures. We also find that PCDH7 overexpression reduces synaptic NMDA receptor currents. These data show that PCDH7 can regulate dendritic spine morphology and synaptic function, possibly via interaction with the GluN1 subunit.

## Introduction

NMDA receptors (NMDARs) are a subclass of glutamate-gated ion channels that are heterotetramers consisting two GluN1 subunits and two GluN2A-D/GluN3A-B subunits^1,2^. NMDARs are critical modulators of synapse formation and synaptic transmission. Dysfunction of NMDARs (hyperactivity or hypofunction) have been linked to various central nervous system (CNS) diseases^3^, including epilepsies^4^, schizophrenia^5^, and Alzheimer’s disease^6–8^. Each NMDAR subunit contains an extracellular N-terminal domain (NTD), ligand binding domain (LBD), transmembrane domain (TMD) and a cytoplasmic tail^9^. The N-terminal domain (NTD) of GluN1 has been reported to regulate NMDAR subunit oligomerization and assembly^10^. Functionally, GluN1 NTD is involved in modulating NMDA receptor gating and pharmacological properties^11^. EphB receptors have been reported to bind GluN1-NTD and regulate excitatory synapse formation via this interaction^12^. Other binding partners of GluN1-NTD remain to be discovered.

The cadherin superfamily of cell adhesion molecules consists of more than 100 members^13^. Protocadherins comprise the largest family within the cadherin superfamily, and consist of two major groups, depending on if the genes are clustered on the chromosome: clustered protocadherins and nonclustered^14^. Non-clustered protocadherins (PCDHδ family) are predominantly expressed in the nervous system, and multiple members from this group have been shown to modulate axon/dendrite structure and function and have been reported to be possibly related to human neurological disorders^15–17^. For instance, PCDH8 and PCDH10 (δ2 subfamily) co-localize with PSD95 and regulate dendritic spine density^18,19^. PCDH9 (δ1 subfamily) has been linked to autism spectrum disorder^20^, PCDH17 (δ2 family) to schizophrenia^21,22^, and PCDH19 (δ2 family) to epilepsy in females with mental retardation (EFMR)^23^.

PCDH7 belongs to the PCDHδ1 subfamily and is also called BH (brain, heart)-protocadherin because of its predominant expression in brain and heart^24^. In the brain, based on the RNA-seq data from sorted cells, PCDH7 is highly expressed in neurons and astrocytes^25^. Despite the expression pattern, most studies on PCDH7 have focused on its role in cancer^16^. In recent years, a few studies have implied the role of PCDH7 in brain function: MeCP2 can bind to the promoter region of PCDH7 and down regulate its mRNA level, suggesting a possible link between PCDH7 and Rett syndrome^26^; genome-wide association studies (*GWAS*) show that PCDH7 is linked to epilepsies, is a risk factor for shorter sleep^27^ and is associated with antipsychotic treatment response in schizophrenia patients^28^. On the molecular level, in a proteomic analysis of synaptic clefts, PCDH7 was found in the excitatory synaptic cleft^29^. All the data point to a potential role of PCDH7 in the brain and perhaps in synapses, but the underlying mechanisms remain to be elucidated.

Here we discover that PCDH7 is a potential binding partner of GluN1-NTD via an unbiased screen. We find that *Pcdh7* gene expression is colocalized with *Grin1* in neurons and find PCDH7 enriched in postsynaptic densities (PSDs). When overexpressed, PCDH7 causes “collapse” of dendritic spines and reduces NMDAR currents. In contrast knockdown of PCDH7 results in elongation of spines. Taken together, our findings reveal that PCDH7 is a synaptically localized, GluN1-interacting protein that regulates synapse morphology and function.

## Results

### Identification of PCDH7 as a potential GluN1 interactor

**Figure 1:**
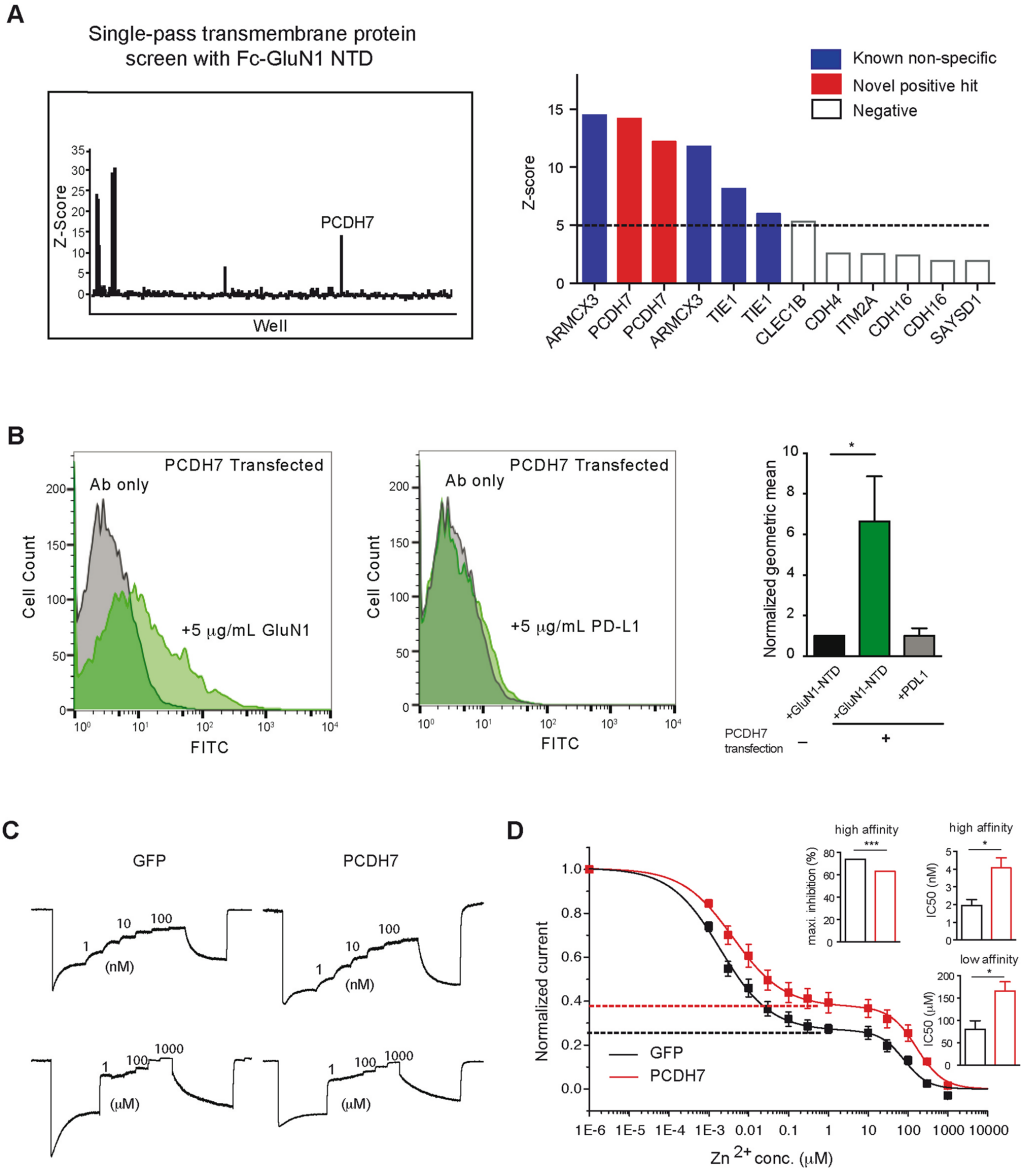
Unbiased screen for GluN1-NTD binding partners. (**A**) left, one representative plate showing results from expression cloning screen with a library of ~ 1,500 single pass transmembrane proteins testing for binding to the purified NTD of GluN1 tagged with human-Fc. The screen was run with duplicate plates for all ~ 1,500 proteins. The *x*-axis shows 384 wells from a single representative plate. PCDH7, the only specific hit is indicated (the other peaks are from Fc receptors or other non-specific binders). Right, top ranked proteins based on z-score, from two repeated runs, excluding Fc receptors. Solid bars represent proteins with a z-score higher than the cut-off of five found in duplicate plates. Red bars represent specific hits and blue bars represent non-specific proteins which exhibit promiscuous binding to numerous baits in this assay. (**B**) FACS plots from 293 cells transiently transfected with PCDH7 and incubated with 5 µg/mL of purified GluN1-NTD-Fc or PD-L1-Fc control. Graph shows normalized geometric mean of signal from Fc-FITC. Student’s t-tests: Control vs. + GluN1-NTD p = 0.0306, Control vs. + PD-L1 p = 0.9919. (**C**) Sample traces of NMDAR current recorded from CHO cells that express GluN1, GluN2A and either GFP or PCDH7-GFP (GFP tagged at C-terminus). Top, high affinity Zn^2+^ inhibition; Bottom, low affinity Zn^2+^ inhibition. (**D**) Quantification of Zn^2+^ inhibition of NMDAR current from experiments as shown in (**C**). n = 3. Inhibition curve was fitted with Hill equation. Bar graphs show the IC_50_ of high and low Zn^2+^ inhibition and extent of maximum high affinity inhibition of NMDAR current. Dotted lines represent maximal inhibition by Zn^2+^ ( PCDH7: 63.14 ± 0.02%,  GFP: 74.16 ± 0.03%, ***p < 0.001). Data are shown as mean ± SEM. Student’s t-test. *p < 0.05.

To identify binding partners of the GluN1-NTD, we used an unbiased screen of single-pass transmembrane proteins. This library of clones consists of about 1,500 genes (Supplementary Table S1) that were individually expressed in the COS-7 cells. We purified the NTD of GluN1 fused at the C terminus to the immunoglobulin Fc region for use as bait. The purified protein was incubated with the COS-7 cells expressing single-pass transmembrane proteins in two replicate experiments (two independent sets of plates). Binding was assessed by detecting the bait protein with fluorescently labeled antibody that recognizes the immunoglobin Fc. Based on the z-score cut-off of 5, out of the ~ 1,500 proteins screened, PCDH7 was the only specific hit that showed up on replicate plates (Fig. 1A, left, one representative plate). There were 20 cadherins (including *N*-Cadherin) and 29 protocadherins included in the library of this screening and none showed binding to GluN1-NTD except for PCDH7. The only other proteins with significant signal were Fc receptors and ARMCX3 and TIE1 which were all considered non-specific because these proteins appear repeatedly as hits in screens with other unrelated bait proteins (Fig. 1A, right).

To validate PCDH7 as an interacting protein of GluN1-NTD, we transiently overexpressed PCDH7 in HEK293 cells, and added either purified Fc-tagged GluN1-NTD or Fc-tagged PD-L1 as a negative control to the cells. After incubation, we stained the cells with FITC-conjugated Fc antibody and used flow cytometry (FACS analysis) to measure the FITC signal. The intensity of the FITC signal reflected the amount of Fc-tagged protein bound to the cell surface. Consistent with the screening results, GluN1-NTD bound to the cell surface of PCDH7-transfected cells, but not the untransfected cells. In addition, Fc-tagged PD-L1 protein did not bind to the PCDH7 overexpressing cells, suggesting the binding was specific between GluN1-NTD and PCDH7 (Fig. 1B).

We were unable to show association of PCDH7 and GluN1 by coimmunoprecipitation (IP) experiments from CHO cells overexpressing GluN1/GluN2A together with PCDH7, or from mouse brain lysates (data not shown). It is possible that the interaction between PCDH7 and GluN1 is of too low affinity to be detected by these methods, or that the interaction depends on some quaternary structure that is disrupted by detergent solubilization of CHO cells or brain tissue.

### PCDH7 modulates Zn^2+^ inhibition of NMDA current

The NTD of GluN1 subunit is involved in Zn^2+^ inhibition of NMDA current. In the presence of NMDA receptor agonists glutamate and glycine, GluN1/GluN2A receptors exhibit a biphasic Zn^2+^ inhibition curve with IC_50_ values in nanomolar and micromolar ranges for the high affinity and low affinity binding sites, respectively. Deletion of GluN1-NTD results in significantly reduced Zn^2+^ inhibition of GluN1/GluN2 receptors^11,30^. Given that PCDH7 can bind the GluN1-NTD, we tested whether PCDH7 affects Zn^2+^ inhibition using a CHO cell line that stably expresses GluN1 and GluN2A^31^. We transfected these GluN1/GluN2A expressing CHO cells with either GFP or PCDH7 + GFP, and recorded NMDAR currents from the transfected cells in response to application of glutamate/glycine and a series of increasing concentrations of Zn^2+^ ions. The baseline currents from GFP and PCDH7 transfected cells before Zn^2+^ application were comparable (Supplementary Fig. S1). As expected we observed a biphasic Zn^2+^ inhibition curve in GFP transfected cells (Fig. 1C, left), with nanomolar IC_50_ value corresponding to high affinity Zn^2+^ binding sites (Fig. 1C, top) and micromolar IC_50_ value corresponding to low affinity Zn^2+^ binding sites (Fig. 1C, bottom). Expression of PCDH7 modestly but significantly decreased Zn^2+^ inhibition, shifting the Zn^2+^ inhibition dose response curve to the right compared to GFP alone: high affinity IC_50_ shifted from 2 to 4 nM (p = 0.03) and low affinity IC_50_ shifted from 79.5 to 165.3 μM (p = 0.04) (Fig. 1C,D). Overexpression of PCDH7 also reduced the maximal inhibition caused by binding to high affinity Zn^2+^ binding sites (63% vs. 74% inhibition) (Fig. 1D). These results showing an effect on this NTD-dependent modulation of NMDARs support the hypothesis that PCDH7 interacts with GluN1 NTD.

### PCDH7 is present in excitatory and inhibitory neurons in mouse postnatal brain and is enriched in PSD fractions

**Figure 2:**
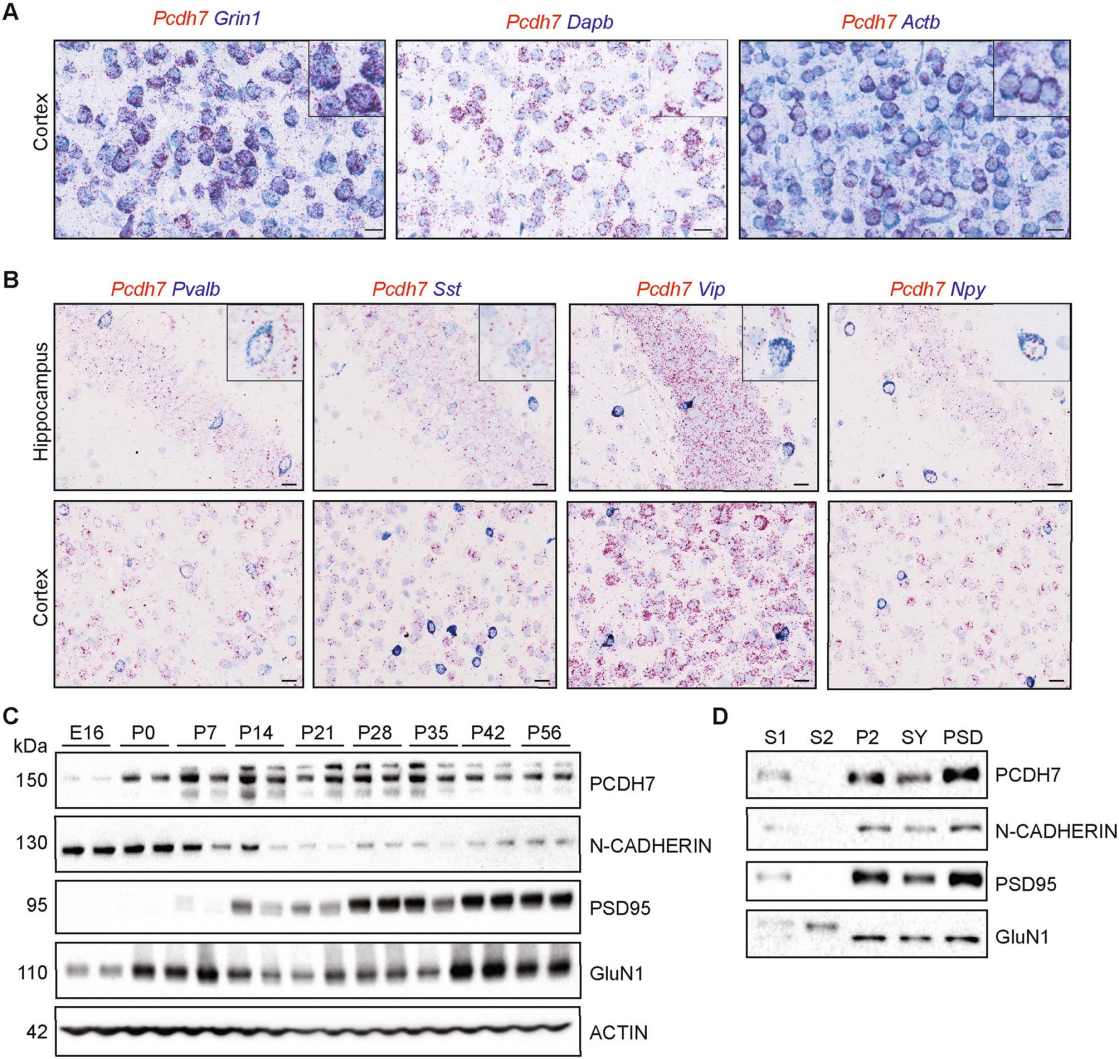
PCDH7 mRNA and protein expression in mouse brain. (**A**) Dual label in situ hybridization of *Pcdh7* (red) and *Grin1* (blue), *Dapb* (bacterial gene as a negative control gene; blue), or *Actb* (universal positive control gene; blue) in P14 mouse brain. Scale bar 20 μm. Insets show zoomed in images. (**B**) Dual label in situ hybridization of *Pcdh7* (red) and *Pvalb*, *Sst*, *Vip*, or *Npy* (blue) in hippocampal CA3 or cortex layer V regions of P14 mouse brain. Scale bar 20 μm. Insets show zoomed in images. (**C**) Timecourse of protein expression in wildtype mouse cortex. Western blots for PCDH7 and other neuronal proteins (*N*-Cadherin, PSD95, GluN1) were run with 50 μg of RIPA lysate collected from forebrain of mouse at indicated developmental timepoints. (**D**) Synaptic fractionation and western blot for PCDH7 and other neuronal proteins was performed on adult mouse brain lysate, *SY* synaptosome fraction, *PSD* postsynaptic density enriched fraction. Western blots were run with 10 μg of lysate from indicated fraction.

Next, we investigated the expression pattern of *Pcdh7* mRNA in mouse brain by in situ hybridization (ISH) with a non-isotopic assay. In adult mouse brain, using dual labeling ISH, we found that *Pcdh7* and *Grin1* were broadly expressed and co-localized in neurons, including in cell layers composed predominantly of excitatory neurons (Fig. 2A). In addition, *Pcdh7* mRNA was also sometimes co-localized with inhibitory neuronal markers such as parvalbumin (*Pvalb*), somatostatin (*Sst*), vasoactive intestinal peptide (*Vip*), and Neuropeptide Y (*Npy*) (Fig. 2B). Thus, *Pcdh7* is expressed in both excitatory and inhibitory neurons.

We performed immunoblotting of mouse forebrain at different ages to examine PCDH7 at the protein level during development. PCDH7 protein increased postnatally, peaked around P14-P28, and continued into adulthood (Fig. 2C). The temporal expression pattern of PCDH7 protein was somewhat similar to the NMDA receptor GluN1, but was almost opposite of *N*-Cadherin. Protein fractionation experiments of mouse forebrain showed that PCDH7 was enriched in the postsynaptic density (PSD) fractions, together with *N*-Cadherin, PSD95 and GluN1 (Fig. 2D). Proteomic studies have reported that PCDH7 is present in the excitatory synaptic clefts^29,32^. Our findings suggest that PCDH7 is concentrated in synapses, where it could interact with NMDARs.

### Knockdown of PCDH7 changes dendritic spine morphology in dissociated neurons

**Figure 3:**
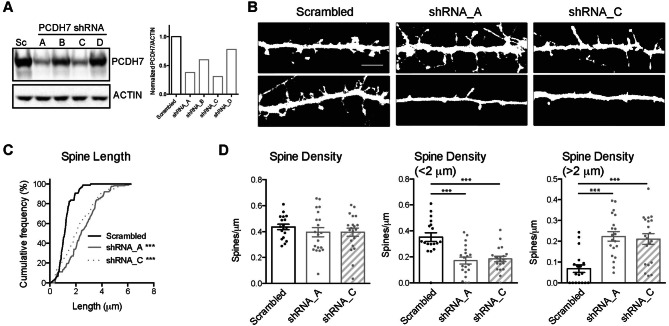
Knockdown of PCDH7 induces morphological change of dendritic spines in dissociated neuronal cultures. (**A**) Western blot from lysate of HEK-293 cells overexpressing rat PCDH7 with either scrambled shRNA or shRNAs targeting rat PCDH7. n = 1. (**B**) Representative confocal images of control or PCDH7 knockdown with two independent shRNAs for 72 h in rat dissociated cortical neuronal cultures at DIV17 (two images per condition). Note the variability of the spine density. Scale bar 5 μm. (**C**) Cumulative frequency of dendritic spine length from 2 independent cultures (n = 18–20 neurons per condition) reveals that PCDH7 knockdown results in significantly longer spines than control. Kolmogorov–Smirnov test: Scrambled vs. shRNA_A ***p < 0.001; Scrambled vs. shRNA_C ***p < 0.001. (**D**) Quantification of spine density. Spines were divided into two groups based on length: less than 2 μm (typical spines) and greater than 2 μm. Total spine density is unchanged with PCDH7 knockdown, but “typical” spine density is significantly decreased after PCDH7 knockdown. Longer or non-typical spine density is significantly increased with PCDH7 knockdown. ***p < 0.001, one-way ANOVA with Tukey’s post-hoc test. All data is presented as mean ± SEM.

In order to understand the physiological function of the endogenous PCDH7, we examined the effect of knocking down PCDH7 in dissociated neuronal cultures. Because the commercially available antibodies we tested could detect overexpressed PCDH7 but were not sensitive enough to detect endogenous protein in neurons, we used heterologous cells to screen for effective PCDH7 targeting shRNAs. By screening four PCDH7 shRNAs using HEK293 cells overexpressing rat PCDH7, we found two shRNAs (shRNA_A and shRNA_C) that suppressed PCDH7 protein levels by more than 60% (Fig. 3A). Dissociated neurons were transfected with either scrambled shRNA, or two distinct shRNAs targeting PCDH7 (together with GFP as a marker protein), and morphology and density of dendritic spines were analyzed. Neurons with reduced PCDH7 either by shRNA_A or shRNA_C showed similar phenotype with longer dendritic spines (Fig. 3B,C). We found that although the total dendritic spine density was not affected by PCDH7 knockdown, there was around 50% reduction in the density of typical spines (length < 2 μm^33^), and about a threefold increase in the density of longer spines (length > 2 μm) (Fig. 3D). While our image resolution precludes a quantitative morphological categorization of spines/protrusions, it is worth noting that a large portion of the longer “spines” appear filopodium-like.

### Knocking down PCDH7 modestly increases dendritic spine length in hippocampal slices with no significant impact on NMDA current

**Figure 4:**
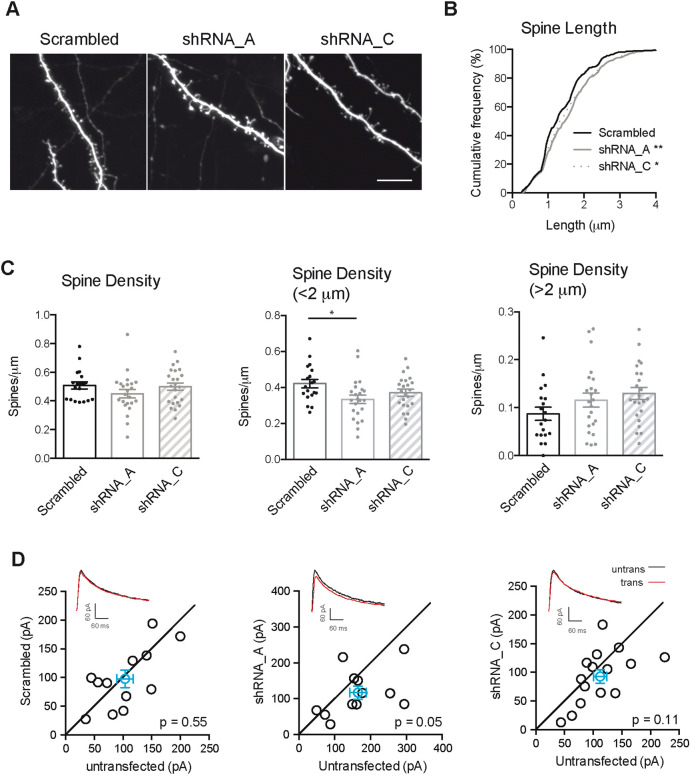
Effects of knocking down PCDH7 in hippocampal slices. (**A**) Representative images of basal dendrites of hippocampal CA1 pyramidal neurons from organotypic slice cultures transfected with scrambled or PCDH7 targeted shRNAs for 5–6 days. (**B**) Cumulative frequency of dendritic spine length. Kolmogorov–Smirnov test: Scrambled vs. shRNA_A **p < 0.01; Scrambled vs. shRNA_C *p < 0.05. (**C**) Quantification of spine density. One-way ANOVA with Tukey’s post-hoc test. All data is presented as mean ± SEM. *p < 0.05. Two independent cultures. n = 19–23 neurons. (**D**) Sample traces and quantification of evoked NMDAR current amplitude from paired transfected (with scrambled or PCDH7 targeted shRNAs) and neighboring untransfected neurons in organotypic slice cultures. Each black circle represents current amplitude of one pair of neurons. Blue circle represents mean amplitude ± SEM. Recordings were done 5–6 days after transfection. 5 independent experiments, n = 12–15 pairs of neurons. No significant effects of knockdown were detected. Paired t test.

To study the role of PCDH7 in a more physiological environment, we used cultured hippocampal slices. Slices were biolistically transfected either with scrambled shRNA or PCDH7 targeted shRNAs. GFP was co-transfected with shRNAs to identify the transfected neurons. Consistent with the data in dissociated neuronal cultures, knocking down PCDH7 in slices resulted in slightly but statistically significantly longer spines without affecting the total spine density (Fig. 4A,B). When the spine density of typical spines (< 2 μm) and longer spines (> 2 μm) were counted separately, there was a trend of decrease in the number of typical spines and increase in the number of longer spines (Fig. 4C). The effect size was smaller in cultured slices compared with dissociated cultures, which is not uncommon in this type of experiment^34,35^, and could reflect stronger compensatory mechanisms in cultured slices.

We performed electrophysiological experiments to test if knocking down PCDH7 affects NMDA receptor function. Stimulation-evoked NMDAR currents were measured at + 40 mV using whole-cell patch clamp from the transfected cell (identified by GFP) and simultaneously from a neighboring untransfected cell. Picrotoxin was added to the solution to block inhibitory current. NMDAR currents were measured 50 ms after stimulation to exclude contamination of AMPAR currents. Comparison of NMDAR currents from transfected and untransfected cells showed that knockdown of endogenous PCDH7 by either of two distinct shRNAs had no significant effect on the amplitude of the evoked NMDA current, although there was a trend towards a slight reduction in current (Fig. 4D). This negative result could mean that endogenous PCDH7 is not limiting in regulating NMDA current, or that the degree of knockdown of PCDH7 was insufficient to cause a phenotype.

### Overexpression of PCDH7 induces morphological changes in dendrites and reduces NMDAR currents in cultured hippocampal slices

**Figure 5:**
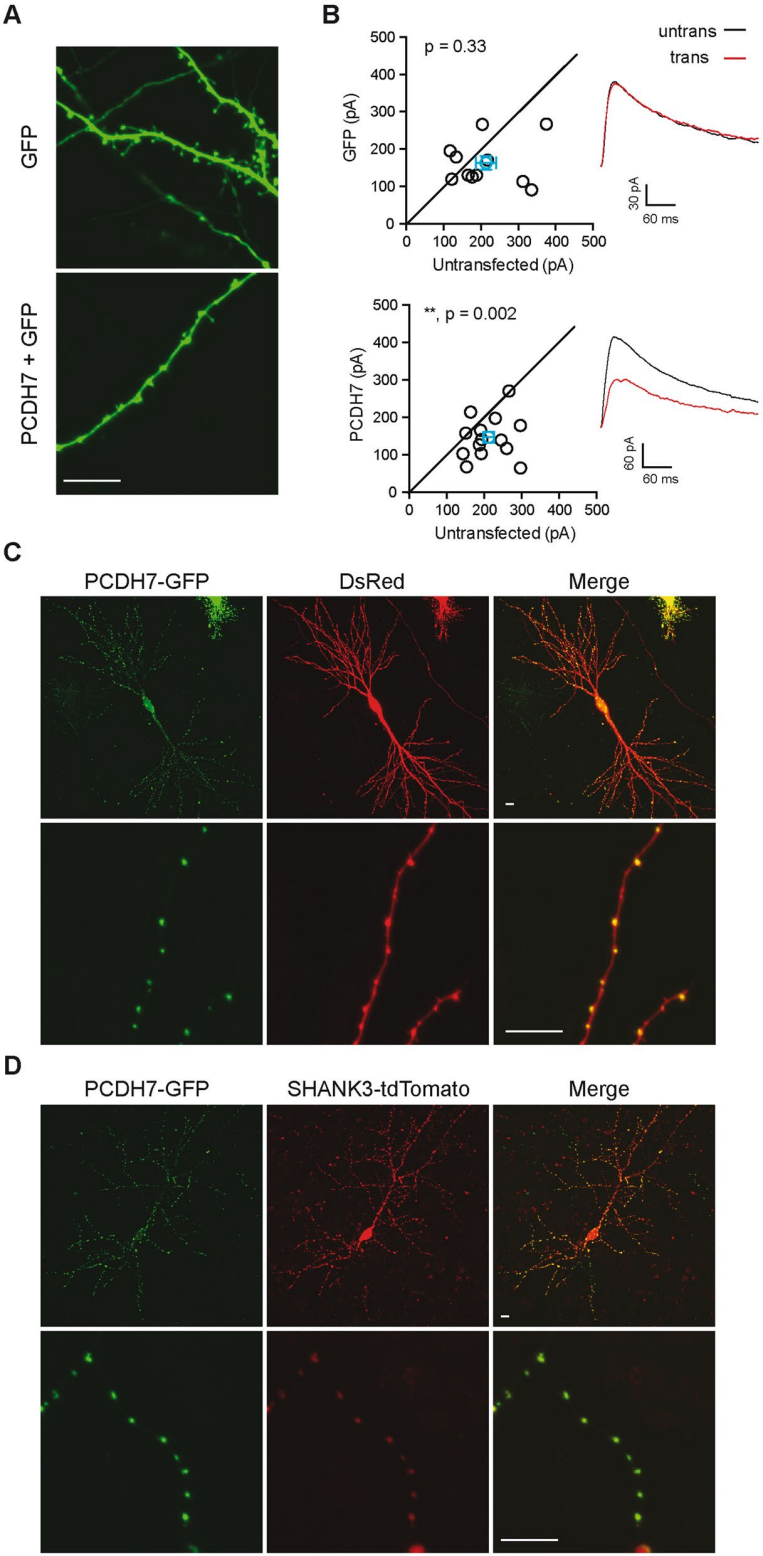
Effects of PCDH7 overexpression in hippocampal slices. (**A**) Representative images of hippocampal CA1 pyramidal neurons overexpressing either GFP or PCDH7 + GFP in cultured hippocampal slices after biolistic transfection for 1 ~ 4 days. Images were taken from basal dendrites. Scale bar 10 μm. (**B**) Sample traces and quantification of evoked NMDAR mediated EPSCs from paired transfected (with either GFP or PCDH7) and neighboring untransfected hippocampal CA1 pyramidal neurons in organotypic brain slice cultures. Each black circle represents current amplitude of one pair of neurons. Blue circle represents mean amplitude ± SEM. Recordings were performed 3–4 days after transfection. 3 independent experiments, n = 11–14 pairs of neurons. **p < 0.01, Paired t test. (**C**) and (**D**) Representative images of hippocampal CA1 pyramidal neurons expressing c-terminal GFP tagged PCDH7 (PCDH7-GFP) with either DsRed (**C**) or c-terminal tdTomato tagged SHANK3 (SHANK3-tdTomato) (**D**). Both whole cell (top) and zoomed in basal dendrites (bottom) are shown. Scale bar 10 μm.

We next investigated the effect of overexpressing PCDH7 in hippocampal slices. We biolistically transfected neurons with either cytoplasmic GFP alone or PCDH7 plus cytoplasmic GFP. As shown in Fig. 5A, neurons overexpressing PCDH7 had dilatations along the dendrites, whereas control neurons had normal spine structures. The morphological change caused by overexpressing PCDH7 looked somewhat opposite to that by knocking down PCDH7: longer spines by knockdown and “collapsed” spines by overexpression. Quantification showed that there were significantly reduced spine/dilatation density and length in PCDH7 overexpressed neurons (Supplementary Fig. S2). We also recorded stimulation-evoked NMDAR currents from transfected and untransfected neurons. Transfection of GFP alone did not affect the NMDAR current amplitude (Fig. 5B, top), whereas overexpressing PCDH7 significantly reduced NMDAR current (Fig. 5B, bottom), which could reflect the collapse of dendritic spines and disruption of synaptic function.

To assess PCDH7 protein localization, we generated a PCDH7 construct tagged with GFP at its C-terminus. We co-transfected this GFP tagged PCDH7 with DsRed as a fill to show neuron morphology (Fig. 5C). Similar to cytoplasmic GFP (Fig. 5A) the DsRed signal showed the presence of multiple dendritic dilatations in neurons transfected with PCDH7 (Fig. 5C, middle). The PCDH7-GFP signal was found in a punctate pattern and was specifically localized to those dilatations (Fig. 5C, left). To get more insight into the dilatations induced by PCDH7 overexpression, we co-transfected GFP tagged PCDH7 with tdTomato-tagged SHANK3, a postsynaptic scaffolding protein, in cultured hippocampal slices. In PCDH7-expressing neurons, SHANK3 had a punctate expression pattern and was highly enriched in those dilatations and strongly co-localized with PCDH7 (Fig. 5D), suggesting that these dilatations might be synapse containing structures. Furthermore, we performed PCDH7 overexpression experiments in dissociated neuronal cultures so that we could stain for endogenous synaptic proteins. Overexpressed PCDH7 was localized to the dendritic dilatations and was partially colocalized with the presynaptic marker synapsin and postsynaptic marker PSD95 (Supplementary Fig. S3A,B). The functional aspect of these structures and how the dendritic morphology change alters NMDAR current remains to be characterized.

## Discussion

Although GluN1 has been studied extensively, little is known about protein interactions mediated by its NTD. Here via an unbiased screen of ~ 1,500 single pass transmembrane proteins, we identified PCDH7 as a potential interactor. PCDH7 is predominantly expressed in the brain, and has been genetically associated with multiple neurological disorders^26–28^. Our *ISH* and biochemical data show that PCDH7 is expressed in both excitatory and inhibitory neurons and the protein is enriched in PSD fractions, which is consistent with the existence of PCDH7 in excitatory synaptic clefts^29^, and supports the hypothesis that PCDH7 may play a role in synapse formation and/or function.

Both overexpression and knockdown of PCDH7 significantly changed the morphology of dendritic structures. Overexpression of PCDH7 resulted in collapse of spines and abnormal dendritic swelling in which PCDH7 and synaptic proteins like SHANK3 were concentrated. In addition, when we transfected cultured hippocampal slices with GFP tagged PSD95, together with PCDH7 and DsRed, overexpressed PSD-95 was also localized to the dendritic dilatations (Supplementary Fig. S3C), consistent with our finding that PCDH7 interacts with GluN1 and the well documented observation that PSD95 and Shank protein colocalize with NMDA receptors at synapses^36,37^. This disruption of dendritic structure was accompanied by a reduction of NMDAR synaptic current. One possible hypothesis is that overexpression of PCDH7 might result in decrease of surface GluN1. Future study with a GluN1 antibody that is suitable for live staining of surface GluN1 would help clarify the mechanism underlying the reduced NMDAR current. On the other hand, knockdown of PCDH7 resulted in longer spines/protrusions. In dissociated neuronal cultures, significantly more of dendritic protrusions exceeded the typical spine length (2 μm), and some of them are filopodia-like, reminiscent of the report showing the elongation of dendritic protrusions after blockade of classic cadherins^38^. Since dendritic filopodia usually represent transient dynamic structures that may turn into spines, this phenotype suggests that there might be more immature synapses, or higher spine motility in PCDH7-knockdown neurons, pointing to a stabilization role of PCDH7 in spines. Time-lapse live imaging of the neurons would help clarify these questions. The elongation of the spines was also significant in cultured slices, but the effect size was smaller, which could be due to either the difference in knockdown efficiency or difference in resilience to partial loss of PCDH7 in a more physiological environment. Theoretically, rescue experiments with shRNA-resistant PCDH7 would provide additional evidence supporting the specificity of the knockdown phenotype. However, as described above, overexpressing PCDH7 had strong effect on dendritic spine morphology in neurons, even with 10% of the amount of DNA normally used for transfection (data not shown). We believe that the effects of overexpression of the shRNA insensitive PCDH7 would overwhelm the effects of knockdown and thus give inconclusive answers. On the other hand, the observations that two distinct shRNAs both induced elongation of spines in dissociated neurons and slice cultures and that overexpression of PCDH7 caused the somewhat opposite “collapse” of spines suggests that the PCDH7 targeting shRNAs effects were most likely specific.

Knockdown of PCDH7 did not affect NMDAR current in our experiments despite changing morphologies of dendritic spines. Since we were only recording from transfected neurons while stimulating presynaptic fibers impinging on those cells, and transfection was sparse in slice cultures, we were only evaluating the potential function of *cis* interaction of PCDH7 with NMDARs or other protein(s) in the postsynaptic site. Moreover, PCDH7 has been reported to bind to phosphatase PP1α via its cytosolic region CM3^16^, and PP1α plays an important role in synaptic plasticity^39^. Therefore, PCDH7 might play a more important role in regulating synaptic plasticity than basal synaptic transmission. On the other hand, overexpression of PCDH7 changed Zn^2+^ inhibition sensitivity of NMDA current in a heterologous system, supporting a role of PCDH7 in direct modulation of NMDAR function and perhaps synaptic function under certain circumstances. Besides neurons, PCDH7 mRNA is also highly expressed in astrocytes. Previous reports have shown that astrocytic γ-Pcdhs are important for synaptogenesis and dendrite complexity^40^. Whether astrocytic PCDH7 plays a role in regulating synapse formation awaits to be examined.

While our data do not distinguish whether the effects of PCDH7 on synaptic function are due to the interaction with GluN1 that we identified, the demonstration of this potentially physiologically relevant interaction, and the characterization of synaptic effects of PCDH7 should provide the basis for future studies exploring mechanisms NMDAR protein interactions and regulation of synaptic function.

## Methods

### Animal use

All rodent experiments were approved by the Genentech Animal Care and Use Committee and followed the National Institutes of Health *Guide for the Care and Use of Laboratory Animals*.

### DNA constructs

Human PCDH7 and PCDH7-GFP were in pRK5 vector; EGFP (GFP), DsRed and rat *Pcdh7* were in pCAGGS vector; SHANK3-tdTomato was in pcDNA vector; PCDH7 rat shRNA constructs (both scrambled and targeted) were ordered from Origene.

### Antibodies and reagents

PCDH7, Abcam ab139274 clone 2G6, mouse monoclonal mouse antibody used for immunoblot at 1:1,000 and FACS at 1:50; NMDAR1, Novus NB300-118 clone R1JHL, mouse monoclonal antibody used for immunoblot 1:1,000; NMDAR1, Cell Signaling 5704 clone D65B7, rabbit monoclonal antibody used for FACS 1:50; PSD-95, Cell Signaling 3409 clone D74D3, rabbit monoclonal antibody used for immunoblot 1:1,000; *N*-Cadherin, Cell Signaling 4061, rabbit polyclonal antibody used for immunoblot 1:1,000; Actin, Cell Signaling 5125 clone 13E5, rabbit monoclonal HRP conjugated antibody used for immunoblot 1:2,000; Cy5, Invitrogen 81-6716, rabbit anti-mouse IgG conjugated to Cy5 secondary antibody used for FACS 1:50; Fc-FITC, Novus NBP1-74956, goat anti-human IgG conjugated to FITC secondary antibody used for FACS 1:50.

### Protein expression and purification

Human GRIN1 (D23-Q393) fused to the Fc portion of human IgG1 was cloned into a vector derived from pAcGP67A (BD biosciences) that included a gp67 signal sequence for protein secretion in insect cells. hGRIN1-Fc was expressed for 72 h in Sf9 cells at a multiplicity of infection of 0.5. It was affinity purified using a MabSelect Sure column (GE Healthcare), and subsequent purification was done by size-exclusion chromatography with a Superdex 200 column (GE Healthcare) according to the manufacturer’s instructions^41^.

### Single-clone expression cloning screen for binders of GluN1-NTD

A clone library containing ~ 1,500 genes representing single-pass transmembrane proteins was assembled with the goal of having each gene represented by at least one full-length sequence verified cDNA clone. Clones were obtained from in-house collection (Genentech) or purchased from external vendors. Complete list of genes is provided in Supplementary Table S1. Integrated automated systems were used for clone library preparation, transfection, binding and detection and high-content imaging. DNA were arrayed in 384-well imaging plates (Aurora Biotechnologies, 32421-PDL) at 60 ng per well, stored at − 80 °C and thawed on the day of transfection. Fugene 6 (Promega) transfection reagent was prepared in Opti-MEM (Invitrogen) at a ratio of 3:1 ratio of Fugene:DNA according to the manufacturer’s manual and incubated for 5 min. Then it was mixed with DNA in 384-well plates for 15–45 min. COS7 cells (ATCC) were added into 384-well plates at a density of 3,000 cells per well and incubated for 48 h. On day of interaction screen, transiently transfected cells were first washed three times with PBS and then blocked with 1% BSA (in PBS) for 1 h. After block, the cells were incubated with GluN1-NTD-Fc protein (bait) (10 μg/mL) for 1 h at 4 °C. After washing, cells were fixed with 4% paraformaldehyde (in PBS) for 30 min at room temperature and incubated with Alexa Fluor-488 labeled anti-human IgG (Invitrogen) antibody (10 μg/mL) for 1 h. Protein binding was represented by Alexa Fluor-488 fluorescent signal and the IsoCyte (Molecular Devices) was used to acquire and analyze the signal for entire plate. Z-score (the number of standard deviations from the mean) was calculated and candidate hits were identified based on a z-score threshold of > 5. Promiscuous and highly nonspecific binders that appear in multiple screens were not considered hits. Genes encoding receptors with affinity for the Fc region of immunoglobulin served as positive binding controls for Fc-tagged Bait proteins.

### Non-isotpic in situ hybridization

Non-isotopic in situ hybridization (ISH) was performed on 4 µm formalin-fixed paraffin embedded tissue sections on a Tecan platform equipped to carry out non-isotopic ISH. QuantiGene^®^ ViewRNA ISH tissue assay kits (Affymetrix) for single and 2-plex probes were used following the manufacturer's protocol^42,43^. Deparaffinization was carried out on a Leica XL stainer and after the boiling step in a Thermo Scientific PT module the slides were briefly dipped in ethanol and dried before assembling the ISH chamber for the Tecan platform. A probe set to Bacillus subtilis dihydropicolinate reductase (dapB) (L38424) was used as a negative control. A probe set to murine ACTB (NM_007393.1) was used as a positive control. Gene-specific probe sets for detection of PCDH7 (NM_018764), GRIN1 (AF146569), PVALB (NM_013645), SST (NM_009215), VIP (NM_001313969), and NPY (NM_023456) were used on tissue samples. Horseradish peroxidase (HRP)-conjugated label probe was used, followed by tyramide signal amplification (TSA) to increase sensitivity (Perkin Elmer). Briefly, TSA plus DIG stock solution (digoxigenin) was diluted 1:50 in 1 × Plus Amplification Diluent, applied to sections, and incubated for 10 min at room temperature. This was followed by incubation with anti-DIG-AP (Roche) diluted 1:500 in TNB blocking buffer with 4% lamb serum (Gibco) for 30 min at room temperature. Vulcan Fast Red and Ferangi Blue chromogens (Biocare Medical) were used for chromogenic detection. Hybridized target mRNAs were visualized using bright field microscopy.

### Time-course of protein expression in mouse cortex

Homogenization of mouse forebrain was performed in ice-cold RIPA buffer with protease and phosphatase inhibitors^44,45^. Briefly, hippocampi were dissected in ice-cold PBS, homogenized, spun to remove debris, snap frozen in liquid nitrogen, and stored at − 80 °C until blotting. Protein extracts of 50 μg were run on SDS-PAGE followed by immunoblotting.

### Primary neuronal culture

As described previously^46^, embryos from timed pregnant Sprague–Dawley rats were removed at E18 and decapitated into cold HBSS/HEPES buffer. The cortex including hippocampus was dissected and placed into 5 mL of HBSS/HEPES buffer. Tissue was washed 4 times before digestion at 37 °C for 20 min with papain (0.5 mL at 200 U/mL into 4.5 mL HBSS/HEPES buffer with tissue). Tissue was washed four times with warm HBSS/HEPES buffer and once with neurobasal media. Dissociated tissue was triturated and passed through a 70 μm filter before plating on poly-d-lysine/laminin coated plates or coverglass with neurobasal media supplemented with B27, Glutamax, and Pen/Strep. Cells were plated at a density of 120,000 cells/well in 24 well plates with coverglass for imagining. Media was exchanged 100% 3–5 h after initial plating and then exchanged 50% once per week.

### Transfection, immunofluorescence, and quantification of primary neuronal cultures

Neuron transfection and staining were done as previously described^46^. Primary neuronal cultures were transfected between DIV12-14 with lipofectamine 2000. Neurons were transfected by incubating cells with 2 μL lipofectamine, 400–1,000 ng DNA or shRNA, and 500 μL neurobasal for 45 min. Cells were fixed 1–3 days post-transfection, as indicated in figure legends, using 4% paraformaldehyde in PBS for 12 min at room temperature. Cells were permeabilized and blocked using GDB buffer (0.2% gelatin, 0.5% Triton-X, 0.8 M NaCl in PBS) and incubated with primary antibodies overnight at 4 °C. Antibodies were visualized using Alexa dye-conjugated secondary antibodies. Images were acquired by a single experimenter blinded to transfection or treatment using a Zeiss LSM780 laser scanning confocal microscope with a 20× or 100× oil objective (0.5 μm confocal z-step size). For spine quantifications, images were quantified by a single experimenter blinded to transfection or treatment using ImageJ software. Image z-stacks were collapsed into maximum projections and thresholded prior to analysis. Dendritic spines from secondary dendrites were analyzed. Spine density was calculated by counting the total number of dendrite protrusions using the GFP channel along a ~ 20 μm segment of dendrite. Spine length was also measured. The cumulative frequency of spine length was calculated using Prism6 software.

### Organotypic hippocampal slice culture, transfection and imaging

Organotypic slices were cultured from P7 Sprague Dawley rats. Biolistic transfection with gene gun (Biorad) was done at DIV3-7^47^. When co-transfecting PCDH7 or shRNA with GFP, gold particles (1.6 μm) were coated with PCDH7 or shRNA with GFP at the ratio of 9:1. Live imaging of transfected neurons was taken using Olympus confocal microscope, with a water-immersion 40× objective (NA 0.8, Olympus) in ACSF solution (see below) and data analysis was done by MATLAB software. For both the experiments and data analysis, the investigator was blinded to the conditions.

### Electrophysiology

For patch clamp recordings of hippocampal CA1 neurons from organotypic slices^48^, the recording solution was oxygenated artificial CSF (ACSF) containing the following (in mM): 127 NaCl, 2.5 KCl, 25 NaHCO_3_, 1.25 NaH_2_PO_4_, 25 glucose, 4 MgSO_4_, and 4 CaCl_2_, along with 100 μM picrotoxin and 2 μM 2-chloroadenosine. NMDAR mediated EPSCs were recorded at holding membrane potential at + 40 mV in the presence of NBQX (10 μM) or measured 50 ms after stimulation artifact. Pipette solution contained the following (in mM): 140 Cs methanesulfonate, 10 HEPES, 2.5 MgCl2, 10 EGTA, and 5 QX-314 Cl.

Doxycycline-inducible CHO cell line that express GluN1 and GluN2A was as previously described^31^. CHO cells were transfected with either GFP or PCDH7-GFP using lipofectamine 2000. Whole-cell recordings from transfected CHO cells were performed using Dynaflow Resolve rapid solution exchange system (Cellectricon) as described^31^. The pipette solution contained (in mM) 120 CsF, 10 NaCl, 2 MgCl2, and 10 HEPES, adjusted to pH 7.2 with CsOH. The extracellular solution to test Zn^2+^ inhibition contained (in mM): 140 NaCl, 2.8 KCl, 1 CaCl_2_, and 10 HEPES, adjusted to pH 7.4 with NaOH. In order to make solution containing free Zn^2+^ (in nM): 1, 3, 10, 30, 100, 300, 1,000, 10 mM Tricine was added to buffer Zn^2+^, and the added Zn^2+^ concentration was (in μM) accordingly: 0.26, 0.78, 2.6, 7.8, 26, 77.5, 254^30^. To make solution containing free Zn^2+^ higher than 1,000 nM, the corresponding Zn^2+^ was directly added to the extracellular recording solution without tricine. 100 µM glutamate and 50 μM glycine were added to the faxt exchanging solution to elicit the currents. Currents were recorded at holding potential of -50 mV.

### Knockdown of PCDH7

Rat PCDH7, along with shRNA against rat PCDH7 were transfected into HEK293 cells for 72 h. Four potentially PCDH7-targeted shRNA constructs were tested as well as a control scrambled shRNA. To determine the extent of PCDH7 knockdown, cell lysates were blotted for PCDH7 or ACTIN loading control. PCDH7_A and PCDH7_C were the most effective and chosen to test on primary neuronal cultures.

### Synaptic fractionation

Mouse forebrain homogenates were fractionated via sucrose gradient^45,49^. Briefly, the forebrain was dissected and mechanically homogenized using a glass–Teflon homogenizer in 5 mM HEPES pH 7.4, 1 mM MgCl_2_, 0.5 mM CaCl_2_ with phosphatase, and protease inhibitors. Samples were spun for 10 min at 1,400×*g* to clear non-homogenized tissue, and an aliquot of the supernatant was taken as S1. Next, the supernatant was spun for 10 min at 13,800×*g* and the pellet was resuspended in 320 mM sucrose, 6 mM Tris. Aliquots of the supernatant S2 and resuspended pellet P2 were collected. The P2 resuspension was further fractionated via a stepwise sucrose gradient, 1,200 mM sucrose, 1,000 mM sucrose, 850 mM sucrose, and P2 in 320 mM sucrose. The gradient was spun at 82,500×*g* for 120 min. The synaptosome fraction (SY) sits between the 1,200 mM and 1,000 mM gradients. An aliquot SY was collected from this fraction and the rest was treated with 0.5% triton for 15 min rotating at 4C. Sample was spun at 200,000×*g* for 20 min and supernatant was collected as final PSD fraction. 10 µg of protein per fraction was loaded onto SDS-PAGE followed by immunoblotting.

### FACS binding assay

HEK293 cells transfected with full length human PCDH7 using lipofectamine 2000. Approximately one million cells in a final volume of 500 μL were used for each experiment. Cells were washed with PBS and then trypsinized to create a single cell suspension. Cells were washed 1× with FACS Buffer (PBS, 0.2% BSA, 0.09% Sodium Azide) and then blocked for 45 min at 4 °C in 1% BSA. After washing cells 3× with 1% BSA, cells were incubated in 2 mL volume of 1% BSA with 5 μg/mL of purified protein (GluN1-NTD-Fc, or PD-L1-Fc control protein) for 1 h at 4 °C. After incubation, cells were washed 3× with FACS buffer. HEK293 cells were stained for PCDH7 followed by Cy5 to confirm transfection and surface expression. Cells were stained with human-Fc-FITC conjugated to detect if GluN1-NTD-Fc, or PD-L1-Fc was bound to cells. All antibody incubations were performed for 10 min rotating at 4 °C. Cells were washed 3× in FACS buffer before final resuspension in 500 µL. Flow cytometry was done using FACSCalibur (BD Biosciences). The FITC geometric mean was calculated using FlowJo software and normalized first to the background (PCDH7 transfected cells stained for Fc-FITC). The final values are plotted normalized to control (untransfected cells + 5 μg/mL GluN1-NTD-Fc stained for Fc-FITC.

## Supplementary information

Supplementary file1 (XLSX 32 kb)

Supplementary file2 (PDF 7004 kb)

## Data Availability

The datasets generated during and/or analyzed during the current study are available from the corresponding author on reasonable request.
