# Supplementary material for: PCDH7 interacts with GluN1 and regulates dendritic spine morphology and synaptic function
Source: Sci Rep. 2020 Jul 2;10:10951. doi: 10.1038/s41598-020-67831-8 (PMC7331671; doi:10.1038/s41598-020-67831-8)
Supplement: Supplementary file 2 — Supplementary file2 (PDF 7004 kb) [file 41598_2020_67831_MOESM2_ESM.pdf]

Supplementary Information (S1-S3)

PCDH7 interacts with GluN1 and regulates dendritic spine morphology and synaptic function

Yuanyuan Wang[1,2,*], Meghan Kerrisk Campbell[1,2,5], Irene Tom[3], Oded Foreman[4], Jesse E. Hanson[1] and Morgan Sheng[1,6,*]

[1]Department of Neuroscience, Genentech Inc., South San Francisco, CA 94080, USA

[3]Department of OMNI-Biomarker, Genentech Inc., South San Francisco, CA 94080, USA

[4]Department of Pathology, Genentech Inc., South San Francisco, CA 94080, USA

[5]Meghan Kerrisk Campbell's present address: Alkahest Inc., San Carlos, CA 94070, USA

[6]Morgan Sheng's present address: Stanley Center for Psychiatric Research, Broad Institute of MIT and Harvard, Cambridge, MA 02142, USA

[2]These authors contributed equally to this work

[*]Correspondence: wangy111@gene.com (Y.W.), msheng@broadinstitute.org (M.S.)

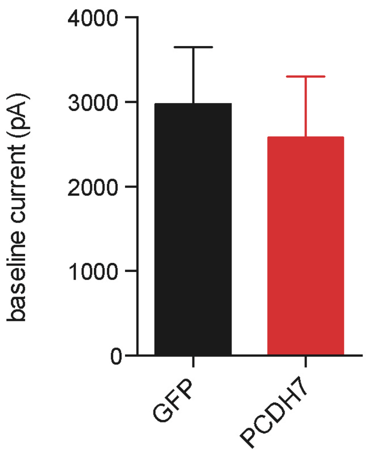

Figure S1. Related to Figure 1D. Quantification of baseline NMDAR current from experiments as shown in Figure 1C.
Data is shown as mean ± SEM. Student’s t-test. n.s.

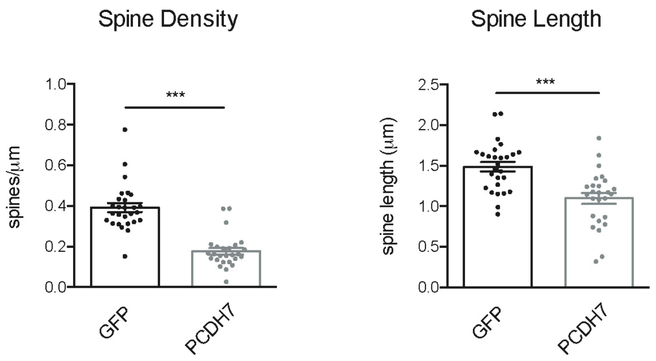

Figure S2. Related to Figure 5A. Quanfication of effects of overexpressing PCDH7 on dendritic spines in hippocampal slices.
Both normal spines and dendritic dilatations are called "spines" in this quantification.
All data is presented as mean ± SEM. Student's t-test. ***, $p < 0.001$. n = 27 for GFP transfected neurons and n = 26 for PCDH7 overexpressing neurons. 4-5 independent cultures.

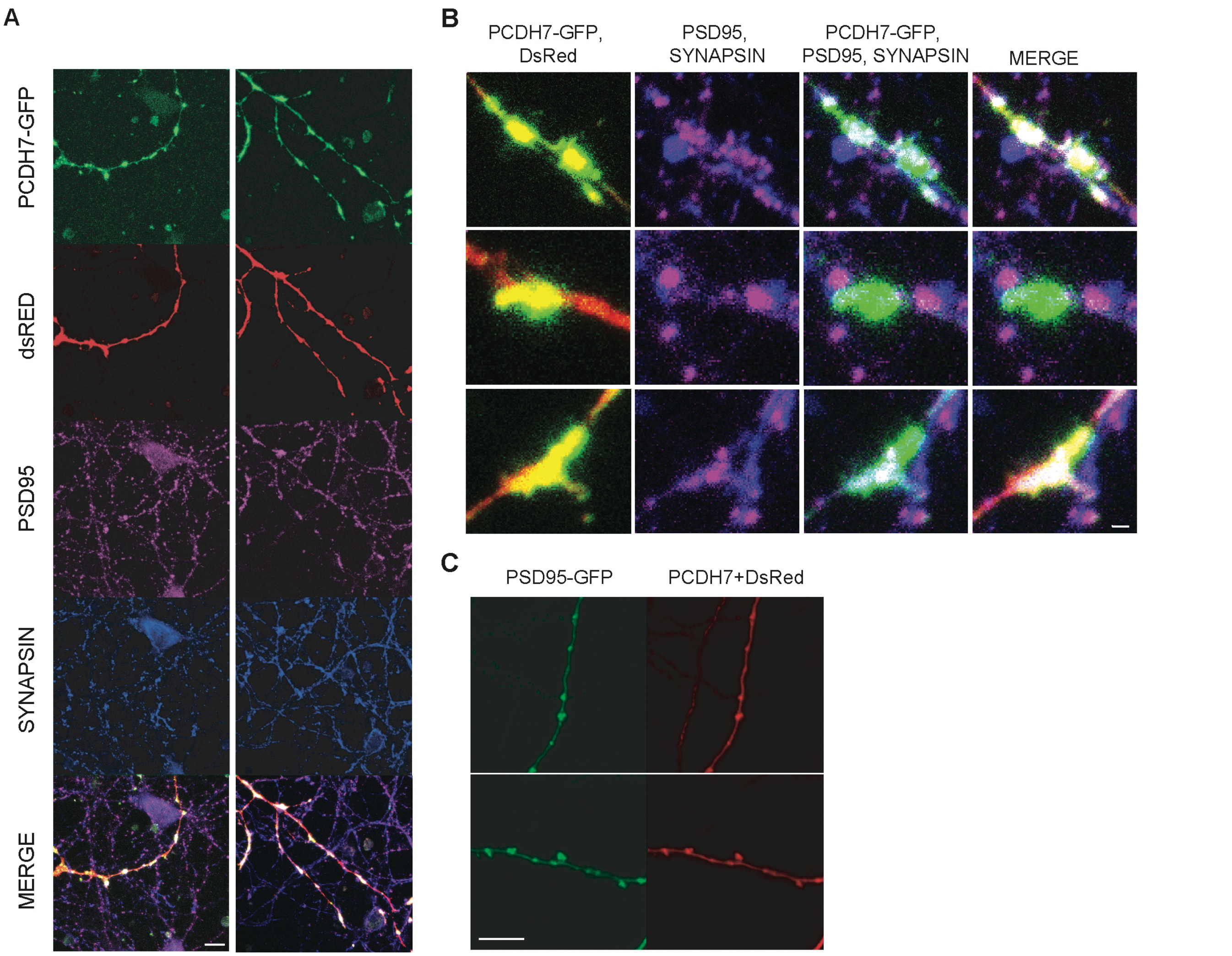

Figure S3. Overexpressed PCDH7 is localized to dendritic dilatations, colocalizes with synaptic markers in dissociated neurons and colocalizes with overexpressed PSD95 in slices.
(A) Neurons transfected with human PCDH7-GFP (green) and dsRED (red) for 24 hours prior to staining for SYNAPSIN (blue) and PSD95 (magenta). Scale bar 10 μm. Two representative images are shown.
(**B**) PCDH7 protein partially colocalizes with and is surrounded by SYNAPSIN (blue) and PSD95 (magenta). Scale bar 0.5 μm. Three representative dendritic sections are shown.
(C) Representative images of hippocampal CA1 pyramidal neurons expressing GFP tagged PSD95 (PSD95-GFP) with PCDH7 and DsRed. Scale bar 10 μm**.**
